# Subgraph Learning for Topological Geolocalization with Graph Neural Networks

**DOI:** 10.3390/s23115098

**Published:** 2023-05-26

**Authors:** Bing Zha, Alper Yilmaz

**Affiliations:** Photogrammetric Computer Vision Lab, Department of Civil, Environmental and Geodetic Engineering, The Ohio State University, Columbus, OH 43210, USA; yilmaz.15@osu.edu

**Keywords:** geolocalization, subgraph, map, graph neural network, motion trajectory

## Abstract

One of the challenges of spatial cognition, such as self-localization and navigation, is to develop an efficient learning approach capable of mimicking human ability. This paper proposes a novel approach for topological geolocalization on the map using motion trajectory and graph neural networks. Specifically, our learning method learns an embedding of the motion trajectory encoded as a path subgraph where the node and edge represent turning direction and relative distance information by training a graph neural network. We formulate the subgraph learning as a multi-class classification problem in which the output node IDs are interpreted as the object’s location on the map. After training using three map datasets with small, medium, and large sizes, the node localization tests on simulated trajectories generated from the map show 93.61%, 95.33%, and 87.50% accuracy, respectively. We also demonstrate similar accuracy for our approach on actual trajectories generated by visual-inertial odometry. The key benefits of our approach are as follows: (1) we take advantage of the powerful graph-modeling ability of neural graph networks, (2) it only requires a map in the form of a 2D graph, and (3) it only requires an affordable sensor that generates relative motion trajectory.

## 1. Introduction

One of the enduring challenges for the autonomous agent in the field of geoinformatics, computer vision, and robotics is to determine its location in the environment. The concept of location is inherently relative, and one cannot describe the location of an object without providing a reference or map. For instance, the location of a person in a city can be specified by how far away that person is from a building, or the location can be pinned on a map. Without loss of generality, all localization techniques generally provide two basic pieces of information: (1) what is the current position (precise or coarse, 2D or 3D) of the object in some reference or map? (2) what is the orientation (2D or 3D) in that same reference or map? The first could be in the form of Cartesian or geographic latitude and longitude or coarse location such as on a certain road or intersection. The second could be a combination of roll, pitch, and yaw or a compass heading. We define the localization in this article as the act of finding an object’s coarse location against a 2D map. Despite many published studies, localization problems still require further research, especially when the global positioning system (GPS) signal is not available in the presence of tall buildings, jammed signals, or indoors [1].

The human brain is a brilliant information processor and is exceptionally skilled at finding one’s location on a map. Such extraordinary abilities have attracted much attention from neuroscientists seeking to explore and model how the human brain performs this fundamental cognitive task. An early neuroscience study has shown that an internal map of the environment referred to as the “cognitive map” uses a graph representation to locate oneself [2] and navigate to a designated destination [3]. For instance, in vector-based navigation agents can simply find their location on a map based on the distance they traversed and corners they turned [4,5]. Understanding such a process and building computational models is crucial to offer advanced artificial intelligent capabilities to a number of applications, including path planning [6] and navigation [7].

In parallel with the exploration of biological mechanisms for localization and navigation, engineered alternative solutions have also been designed to achieve such functionality. The most commonly used system is the GPS, which was established in the 1970s for outdoor positioning using the constellation of a satellite network [8]. Apart from GPS, traditional relative localization typically utilizes visual or inertial information to simultaneously compute the platform’s pose and 3D environmental structure [9]. Despite these studies, there is still no widely accepted solution for localization in challenging conditions, due to environmental confusers, sensor drifts, multi-path problems, and high computational costs.

Unlike the GPS embedded in devices, our brain’s system accesses location and navigation information by integrating multiple signals relating internal self-motion (path integration) [10] and planning direct trajectories to goals (vector-based navigation) [3,11]. Recent research [11,12] has shown that the mammalian brain uses an incredibly sophisticated GPS-like localization and tracking system of its own to help recognize locations and guide them from one location to the next. One typical method used is called path integration [10], a mechanism of calculating location simply by integrating self-motion information, including direction and speed of movement—a task carried out without reference to external cues such as physical landmarks. Another method suggested representing space as a graph structure in which nodes denote specific places and links are represented as roads between pairs of nodes [5]. The resulting graph reflects the topology of the explored environment upon which localization and navigation can be directly implemented by the graph search algorithm. This paper aims at exploiting characteristics from these two methods together.

With the recent progress in deep learning, especially for graph neural networks (GNN) [13,14,15,16], researchers have shown powerful models that yield expressive embedding of non-Euclidean data and result in promising performances in a variety of tasks [7,17,18]. In this paper, the characteristic of a topological map defined on the non-Euclidean domain makes graph neural network architectures very suitable for topological geolocalization problems.

**Figure 1:**
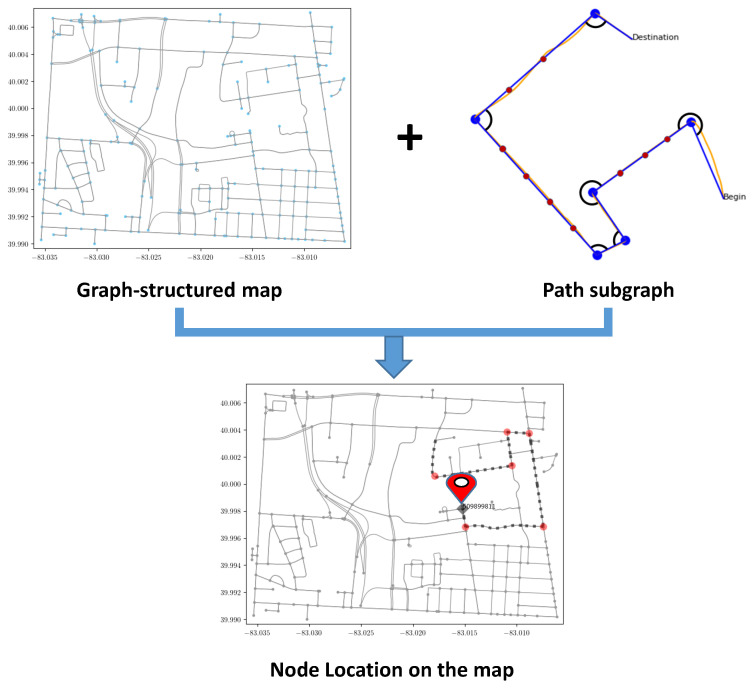
Key Idea: A graph representation of a map is composed of places and their connections on which an object navigates from one place to another. Additionally, object navigation is usually guided by instructions including turns made and distances traversed, based on which a motion trajectory is formed. We are inspired by this observation to generate a possible set of such trajectories and their respective node locations to be used as a dataset to train a graph neural network. The testing in this setup is a path subgraph that is fed into a trained model that in turn outputs the object’s node location on the map.

Inspired by those pioneering research from neuroscience and the progress in graph representation learning, we introduce a topological map-based subgraph learning method for localizing the platform using graph neural networks. As shown in Figure 1, diverse traversed trajectories and corresponding node locations are obtained from a graph-structured map. We then construct a subgraph for the platform trajectories and perform subgraph embedding using graph neural networks. In our application, the unique node ID is the end of the path subgraph and is used as the location label for each subgraph. Therefore, our approach can be divided into two stages. First, the raw motion trajectory is constructed as a subgraph and embedded through a GNN architecture. Second, the embedded subgraph is classified using the fact that each node has a label. In order to demonstrate the effectiveness of the proposed approach, we trained the graph neural network using a large number of possible trajectories generated from the map data and tested the performance on real object trajectories generated using visual inertial odometry, which is the process of estimating the pose and trajectory of a system by fusing measurements from the camera and the inertial measurement unit (IMU) [19]. Note that we use object trajectory throughout the article to indicate any motion trajectories obtained from different platforms, including pedestrians, robots, and vehicles.

The key contributions of this paper are as follows:

- Introduce a novel motion trajectory-based topological geolocalization method using a graph neural network, which combines the benefits of vector-based navigation and the graph representation of a map.
- Design two different subgraph representations for motion trajectories: one is for the encoding direction and the other for encoding both direction and distance by inserting virtual nodes.
- Demonstrate an affordable data collection setup that is used to generate visual-inertial navigation dataset to demonstrate the effectiveness of the proposed method in a practical setting.

## 2. Related Work

**Visual Localization**. A major category of work in the literature is dedicated to the use of images for localization, referred to as visual localization. These methods can be classified into photogrammetric localization [20,21,22,23] and retrieval-based localization [24,25]. The first set of approaches assumes the scene is represented by 3D sparse point clouds, which are commonly generated from structure from motion [26]). Then, the camera pose for a given input image is directly estimated. The training dataset consists of pairs of images and the corresponding camera poses where the camera pose is usually represented by 6-DoF position and orientation. Despite their performance, the photogrammetric pipeline for generating and storing large 3D maps is not trivial and needs a large memory footprint. Another set of methods works by matching a given image to a database of location-tagged images or location-tagged image features. From the hand-craft features such as SIFT [27], bag-of-visual words [28], Fisher Vector [29] and VLAD [30], to the learned features [31,32], all of these approaches struggle to find a good representation robust to changes in viewpoint, appearance, and scale, which is a requirement hard to fulfill in practice. Furthermore, creating an up-to-date image/feature database seems at best costly if not impossible. There is also a potential privacy issue of storing visual descriptors in the database. Our approach mitigates the above deficiencies by using open-sourced 2D maps.

**Probabilistic Localization.** A common form of localization problem is to use sensory readings to estimate the absolute coordinates of the object on the map using Bayesian filtering [33,34,35,36,37]. The authors of [33] presented a Bayesian approach to model the posterior distribution of the position given the prior map, which is considered a classic method commonly adopted in the robotics field. However, this method requires GPS readings and endures a rigorous mathematical model. In more recent studies [34,35], the authors proposed a probabilistic self-localization method using OpenStreetMap and visual odometry where the location is determined by matching with road topology. The authors of [36,37] presented a localization approach based on stochastic trajectory matching using brute-force search. However, all of these methods require the generation and maintenance of posterior distributions, which lead to complicated inference and high computational costs. For interested readers, a more comprehensive reference about probabilistic approaches is given in [38]. In contrast to the above methods, we avoid the complicated probabilistic inference process and propose an intuitive and learning-based approach.

**Topological Localization.** There are a small number of studies closely related to ours that uses topological map and deep learning. Traditional approaches utilize topological road structures and try to match features onto the map using Chamfer distance and Hamming distance [39,40]. Chen et al. [7] proposed a topological approach to achieve localization and visual navigation using several different deep neural networks. However, the method aims at visual navigation problems and is only investigated in a small indoor environment. Wei et al. [41] proposed a sequence-to-sequence labeling method for trajectory matching using a neural machine translation network. This approach was shown to only work well on synthetic scenarios where the input trajectory was synthetically generated with a known sequence of nodes from the map. In [42], the author presented a variable-length sequence classification method for motion trajectory localization using a recurrent neural network, which largely inspired us to employ motion-based data to achieve localization. Zha et al. [43] introduced a topological map-based trajectory learning method and utilized hypotheses generation and pruning strategies to achieve consistent geolocalization of moving platforms where the problems were formulated as conditional sequence prediction. In contrast, this paper focuses on the node localization problem on a topological map based on motion trajectory and develops a subgraph embedding classification model using a graph neural network, which generalizes sequence representation to graph representation and preferably fits the graph-based map structure.

**Vector-Based Navigation.** In neuroscience, much of the literature focuses on studying the mechanisms of animals’ ability to learn maps, as well as self-localization and navigation [2,11,44]. These studies have shown that one typical method used in animals, such as desert ants, is path integration, which is a mechanism in which neurons calculate location by integrating self-motion. Self-motion includes direction and the speed of movement, which inspired us to utilize turning and distance information in this paper. In [5], the authors elaborated on a topological strategy for navigation using place cells [44,45] and metric vector navigation using grid cells [12], from a biological perspective. Our work can be considered as a mixture of topological and vector strategy, where the map is a graph representation, while navigation on the map is vector-based and includes direction and distance.

**GNN on Spatial Data.** The idea of GNN is to generate representations of nodes, edges, or whole graphs that depend on the structure of the graph, as well as any feature information endowed by the graph. The basic GNN model can be motivated in a variety of ways, either from the perspective of a spatial domain [15,46] or a spectral domain [47,48]. Further comprehensive reviews can be found in [13,14,49]. In recent years, the GNN has extended its applications to geospatial data due to its powerful ability to model irregular data structures. For example, the authors of [50] combined the convolutional neural network and GNN to infer road attributes, which overcome the limitation of capturing the long-term spatial propagation of the features; the authors of [51] presented a graph neural network estimator for an estimated time of arrival (ETA), which accounts for complex spatiotemporal interactions and has been employed in production at Google Maps; and the authors of [52] improved the generalization ability of GNN through a sampling technique and demonstrated its performance on real-world street networks. Ref. [53] proposed a GNN architecture to extract road graphs from satellite images.

As summarized above, the localization problem mainly follows the query-to-map paradigm. The representation and usage of query and map are different in the references. To infer the location given the query, numerous methods are proposed. Overall, while the proposed method in the paper has elements in common with the existing works, we develop a novel motion-based query representation and GNN-based learning method, which explicitly distinguish us from the above works.

## 3. Proposed Method

**Figure 2:**
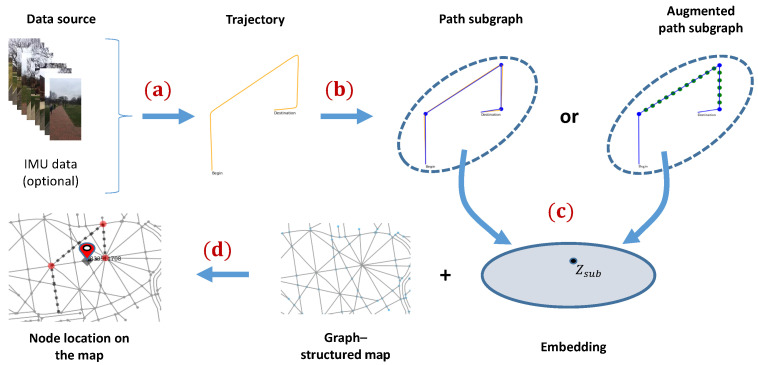
Illustration of the proposed method to achieve topological localization. A forward pass consists of (**a**) acquisition of raw trajectory from visual or/and inertial data source; (**b**) construction of a trajectory graph or augmented trajectory graph by identifying significant turnings in raw trajectories. The augmented trajectory graph encodes both the turns and the distances by inserting virtual nodes; (**c**) each subgraph embedding is obtained by training a graph neural network; and (**d**) classification of subgraph embedding to generate a node label that indicates the final location of the learned map. Note that the training and inference share an identical pipeline except for the subgraph embedding part.

Our approach is built upon two motivations: one is that humans are exceptionally good at self-localization based on observations and a simple “mind map” [2]. The other one is from biological models of navigation that use grid cells [54], which support the calculation of goal-directed vectors, enabling humans and animals to follow directions and distances to a specified target, a process known as vector-based navigation [11]. Combining these two techniques, we develop an approach to infer the location of an object on a map based on the distances traversed and the corners turned. We start with an unknown object location, and as the object traverses the scene, the spatial uncertainty of its whereabouts reduces and a unique location can be estimated based on the conjecture that the motion trajectory would only fit a certain subgraph on the map. To complete this task, we design a learning-based approach using a graph neural network where the input is a traversable path subgraph and the output of subgraph classification is the position of the last node added to the subgraph. During testing, a real object trajectory is represented as a subgraph and “classified” into its location on the map. The overall pipeline of the proposed approach is illustrated in Figure 2.

### 3.1. Problem Formulation

Let a map be defined as a directed graph G=(V,E) with vertices $V=(v_{1},v_{2},\cdots ,v_{n})$ and edges $E=(e_{1},e_{2},\cdots ,e_{m})$, as shown in Figure 1 where *n* and *m* are the numbers of nodes and edges, and each node and edge has a unique id. As the object moves in the environment scene, we fuse visual and inertial sensory data to generate a metric trajectory; the sequence of nodes traversed is converted into a subgraph $G_{s}=\left(V_{s},E_{s}\right)\in G$ in which the attribute of each node and edge is defined as turning angle and road length, respectively. The label of this subgraph is described as the node $v_{i}\in V$ where the last significant turn happens. Therefore, we formulate our topological geolocalization problem as a multi-class subgraph classification problem:

- Input subgraph: $G_{s}=\left(V_{s},E_{s}\right)$, $x_{s}\in R^{|V_{s}|\times d}$, where $|V_{s}|$ is the number of nodes of the subgraph and *d* is the dimension of node attribute;
- Embedding stage: $Z_{s}$ is the embedding of subgraph $G_{s}$ obtained from graph neural network;
- Classification stage: the subgraph embedding $Z_{s}$ is classified into label $y=v_{i}$, $v_{i}\in V$ through fully-connected neural network, where $V=\{v_{1},v_{2},\cdots ,v_{n}\}$ is the output label space and *n* is the number of nodes in the topological map;

### 3.2. Subgraph Representation

**Figure 3:**
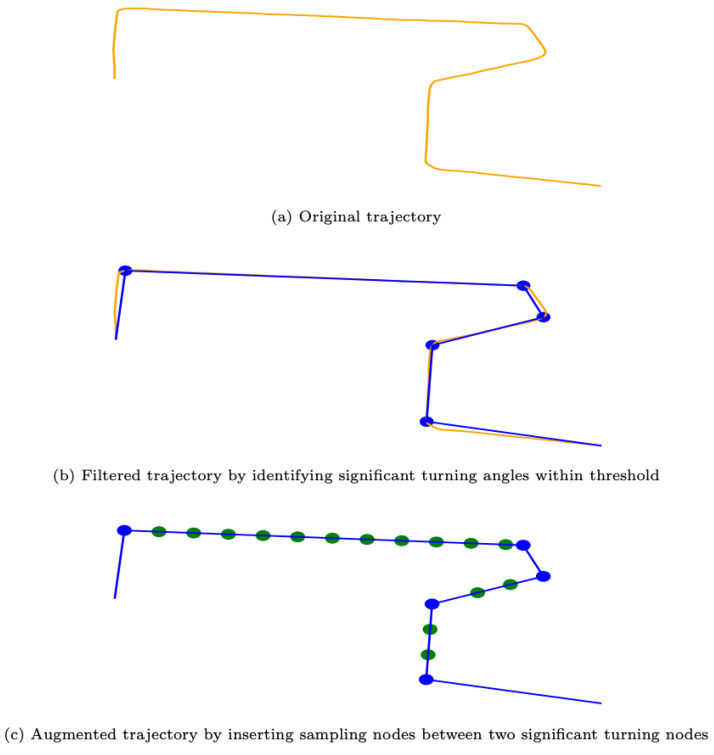
Encode original trajectory into subgraph using two different representations: filtered trajectory graph encodes turning information, and augmented trajectory graph encodes both turning and distance information.

General navigation behavior from source to destination is assumed to form a trajectory as a sequence of turns and distances as shown in Figure 3. Such a pattern conforms to the definition of a “simple path” in graph theory, where the turning place is the node and the distance is the edge leading us to encode the motion trajectory as a path subgraph.

Specifically, the subgraph is defined as a special adjacency matrix where consecutive nodes are always connected, or are otherwise disconnected as expressed in Equation (1):

(1) $$A={\left[a\right]}_{ij}=\left\{\begin{array}{cc} 1, & \mathrm{if}\;\mathrm{vertex}\;i\;\mathrm{and}\;j\;\mathrm{is}\;\mathrm{connected}\\ 0, & \mathrm{otherwise} \end{array}\right..$$

**Figure 4:**
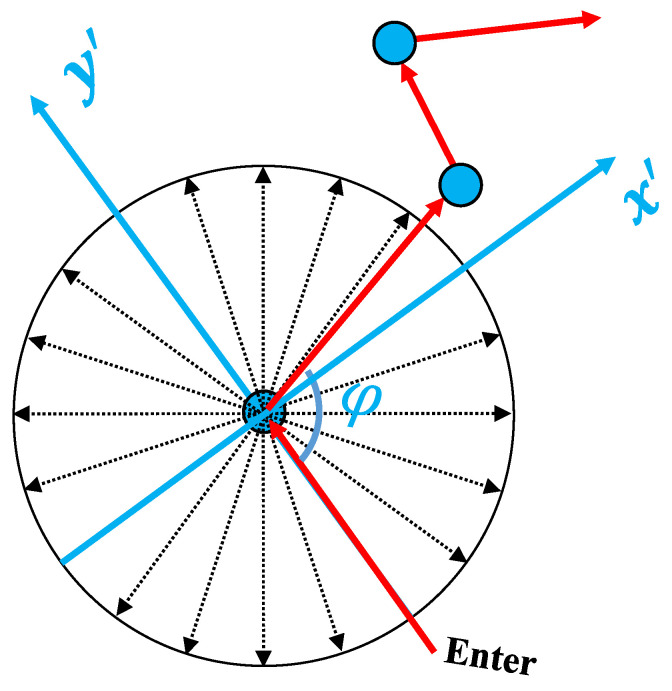
Egocentric coordinate system for angle computation and quantization into discrete angle representation. The illustrated figure uses 20 bins.

In order to ensure the turning angle is rotation-invariant and represents a unique direction, the angle is defined within an egocentric coordinate system [55] that always involves a reference to the current body position as shown in Figure 4. Given a sequence of motions already encoded as a subgraph, three consecutive nodes $\left\{n_{i-1},n_{i},n_{i+1}\right\}$ are used to compute the turning angle by $\theta =\arccos\frac{a\cdot b}{\left|a||b\right|}$ where $a=n_{i}-n_{i-1}$ and $b=n_{i+1}-n_{i}$. Through this formulation, a trajectory with *n* points results in n−2 turning angles, which serve as the node attributes of the subgraph. These turning angles are then quantized into discrete bins so that the representation becomes finite and categorical. The major benefit of choosing quantized input instead of original continuous value is its robustness to noise. Concretely, a turning angle, in reality, could be varied considerably based on different computational methods. However, a discretized angle can still keep the same input and alleviate this problem. Note that the choice of the number of bins is usually dependent on the complexity of the road network and the noise present in motion trajectory.

In real applications, the structure of the subgraph cannot be known except for a given sequence of relative location information. The first strategy to use real data is to identify significant turning locations as “control points”, as shown in Figure 3. Those subsequent “control points” consist of a skeletal graph representing the motion trajectory where the node attribute is set to the computed turning angle. The second augmented representation is created to implicitly incorporate distance information by inserting virtual nodes at uniform distances into the road segment after “control points” are identified as shown in Figure 3. These “virtual nodes” always introduce additional $180^{\circ }$ turning angles which in fact indirectly encode distance information into a subgraph that uses turning angles. The “virtual nodes” design brings two major benefits: (1) we do not need to deal with two different modalities of data (distance and angle) which differ in nature; (2) each node is represented as a location in the map, so the added “virtual node” can make location prediction more precise.

### 3.3. Embedding Stage

In the embedding stage, the aim is to encode the path subgraph into a single representation. Our approach is motivated by a recent work [56] that shows graph neural network architecture can perform subgraph-matching problems by finding nodes in the target graph whose *k*-hop neighborhood contains the query graph. In contrast, our graph is a particular path graph, and we transform the graph-matching problem into a graph classification problem and build a GNN model adapted from GraphSAGE [15] to learn path subgraph representation and perform classification in the graph representation of a map node space, which is suitable for dealing with map-based geolocalization problem.

**Figure 5:**
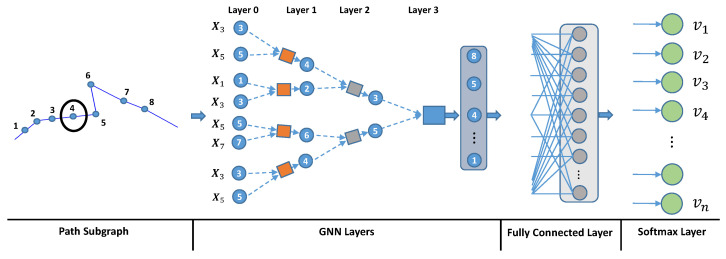
Illustration of embedding trajectory subgraph with a graph neural network layer and a fully connected layer. The GNN layer is used to embed each node’s attribute and integrate it into a single subgraph embedding by graph pooling operation. The fully connected layer and softmax layer serves as a classifier intended to classify subgraph embedding into node space $v_{i}\in E=v_{1},v_{2},\cdots ,v_{n}$.

The defining feature of the graph neural network model is based on a form of neural message passing framework [57] in which vector messages are aggregated between nodes and updated using the neural network structure. We focus on the message passing framework and describe how a subgraph is used as input **$G_{s}=\left(V_{s},E_{s}\right)\in G$** along with a set of respective node attributes $x_{s}\in R^{|V_{s}|\times d}$, to first generate node embeddings that are then transformed into a subgraph embedding. During each message-passing iteration in GNN, as shown in Figure 5, a hidden embedding $h_{v}^{k}$ representing node *v* at layer *k* is updated according to the information aggregated from its previous self-embedding and neighborhood embedding. The update and aggregate operation are expressed as follows:

(2) $$\begin{array}{cc} h_{v}^{k} & =update(h_{v}^{k-1},m_{N(v)}^{k-1})\\ \; & =\sigma (W_{a}h_{v}^{k-1}+W_{b}m_{N(v)}^{k-1}) \end{array}$$

(3) $$\begin{array}{cc} m_{N(v)}^{k-1} & =aggregate^{k-1}\left(h_{u}^{k-1},\forall u\in N\left(v\right)\right)\\ \; & ={\mathrm{MLP}}^{k-1}\left(h_{u}^{k-1},\forall u\in N\left(v\right)\right) \end{array}$$

where both update and aggregate can be any differential function. We adopt an activation function for update and a multi-layer perception (MLP) for aggregate. The superscript denotes the iteration step or layer of message passing; $m_{N_{v}}$ is the “message” aggregated from *v*’s neighborhood $N_{v}$; and $W_{a}$ and $W_{b}$ are the neural network weights that need to be learned.

The initial node embeddings at k=0 are the raw discrete angle representation for all nodes. At each iteration *k* in a GNN layer (e.g., k=3 as in Figure 5), the aggregate function takes as input the set of node embeddings in *v*’s neighborhood $N_{v}$ and generates a message $m_{N(v)}^{k-1}$. The update function combines neighborhood message $m_{N(v)}^{k-1}$ with previous hidden embedding $h_{v}^{k-1}$ to generate a updated node *v*’s embedding at current iteration *k*. After running *k* iterations in GNN, the output of the final layer is used to define the embedding for each node.

In order to generate the subgraph embedding, an extra pooling operation is carried out that allows the GNN to learn a more abstract representation of the subgraph by summarizing the local object motion or its sub-trajectory. As given in (4), a graph-level output $r_{i}$ is computed by adding the node features across the node dimension, which is used as a feature vector for each subgraph:

(4) $$r_{i}=\sum_{n=1}^{N_{i}}x_{n}.$$

### 3.4. Classification Stage

In the classification stage, the final output of GNN is fed into a fully-connected (FC) layer followed by a softmax layer to generate class probabilities. The total number of classes or labels is set to the number of nodes in the entire map denoted as *V* as shown in Figure 5, and hence each label corresponds to a set of input subgraphs of different sizes. The FC layer is simply defined as a linear transformation:

(5) $$o_{i}=f\left(r_{i}\right)=W\;r_{i}+b$$

where $r_{i}$ is a subgraph embedding obtained in the previous stage. W and *b* are the weights and bias parameters needed to optimize. Then, the node class probability is generated by the softmax layer as follows:

(6) $$p^{v}=\frac{e^{W_{\left(v\right)}\;r_{i}+b}}{\sum_{l=1}^{V}e^{W_{\left(l\right)}\;r_{i}+b}}$$

where $p^{v}$ is the probability for node class *v*.

The multi-class classification problem leads us to use the cross-entropy loss function defined in (7) to train the GNN in a supervised way using gradient descent [58]:

(7) $$L=-\frac{1}{V}\sum_{v}^{V}y_{v}log\left(p^{v}\right)$$

where *V* denotes the total number of node classes and $y_{v}$ is the ground-truth for node class *v*.

## 4. Experiments

In this section, we first describe the datasets used in this paper, including map generation; synthetic trajectory generation by graph search algorithm for training; and real trajectories generated by visual inertial odometry and Google Maps for the testing of the proposed approach on three different areas: Ohio State University (OSU) Oval, OSU Campus, and Washington DC, detailed in Section 4.1. Then, the details of implementation and hyper-parameter settings are presented in Section 4.2. In Section 5, we evaluate and verify the proposed method and also compare it with existing approaches to demonstrate its effectiveness. The ablation study is also conducted to evaluate the performance for different nodes of path subgraph and different GNN models. Finally, we discuss the limitations of the proposed method in Section 5.3.

### 4.1. Dataset

The proposed approach is designed to learn the trajectory representation from the synthetic path subgraph and is tested on real-world generated object trajectories. To this end, we introduce the map generation and training data generation and then describe the testing data generation.

#### 4.1.1. Map Generation

As for the acquisition of map for our purpose, we adopt OpenStreetMap (OSM) (www.openstreetmap.org, accessed on 20 May 2020), which is freely accessed online, and the user can download a specific area of interest by manually selecting a bounding box **b** in terms of longitude and latitude, **b** = $\left(lon_{min},lon_{max},lat_{min},lat_{max}\right)$. The obtained map is given in XML format, from which we abstract the file as a directed graph structure where each node represents the place in the map with attributes of its geographic coordinates and each edge denotes different road segments. Thus, an agent can be able to navigate freely on the such map as a graph traversal process forming different graph paths, which will be used as a training dataset in this paper.

#### 4.1.2. Map-Based Trajectory Generation

**Figure 6:**
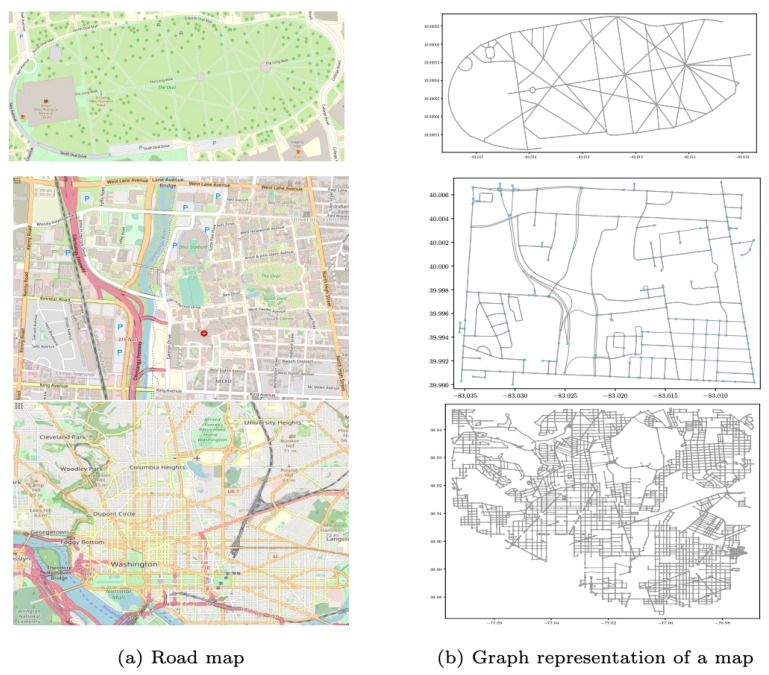
Map graphs: from the top row to the bottom row are small, medium-sized, and larger maps.

The proposed subgraph learning process uses motion trajectories and topological maps in a supervised learning setup. Therefore, we generate a set of possible trajectories associated with labels from the provided map as training data. The possible trajectories are generated using a modified depth-first search algorithm [59] that takes a source node and a target node as its input; the algorithm generates all possible trajectories without repeating edges. In order to limit combinatorial explosion in data generation, the maximum number of nodes that can be traversed is limited to ten nodes assuring that the object moves on shorter routes. Note that the directed graph denoting allowed motion directions can also significantly reduce the complexity of trajectory generation. Three different map sizes are tested: small-sized map (S), medium-sized map (M), and large-sized map (L), as shown in Figure 6 and the map graph information is presented in Table 1.

As mentioned in Section 3.2, two different trajectory representations are generated. The training dataset statistics are summarized in Table 2, where the original training dataset is the sets of trajectories generated from the map; the filtered dataset is for trajectories that only contain significantly large turning angles (30${}^{\circ }$ in this article) as “control points”, and the augmented dataset contains all of the trajectories augmented by inserting virtual nodes. The difference between the “filtered” and “augmented” path subgraph is the number of nodes of the path subgraph and will not change the number of total trajectories. Note that for reducing the training time, we only select part of the nodes in each map as training classes. Each class corresponds to a set of path subgraphs, and the goal of training is to classify these path subgraphs into correct node classes also known as “locations”.

#### 4.1.3. Generating Real Trajectory Data for Testing

**Figure 7:**
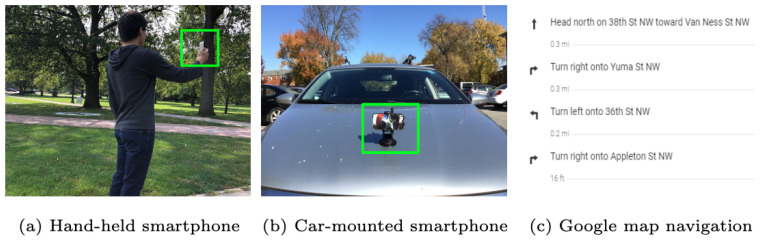
Three ways to collect real trajectory data for testing: the left and medium ones are used for collecting trajectories through visual-inertial odometry in the small- and medium-sized map; the last one uses Google Maps to collect trajectory data in the large-sized map.

The validation of our approach is conducted using trajectories generated by visual-inertial odometry in the small and medium size maps. As illustrated in Figure 7, we used a smartphone to generate trajectories for each map size. The small-sized map uses a walking trajectory, and the medium-sized map uses a driving trajectory. For trajectory generation, open-source MARS Logger [60] was used. This library provides a smartphone application that can collect synchronized video and IMU data. The trajectories are generated by employing the visual-inertial odometry approach [19] and are observed to provide relatively good motion data with metric information. We collected data for 20 different walking traverses while we hold the camera in the forward-looking direction in our hand, and 10 driving traverses using a vehicle-mounted version. As for the large-sized city map, we artificially generated 50 driving routes using the directions provided by Google Maps for specified destinations. This information provided us with a sequence of distances and turns for each navigation route, as shown in Figure 7.

### 4.2. Training Process

**Figure 8:**
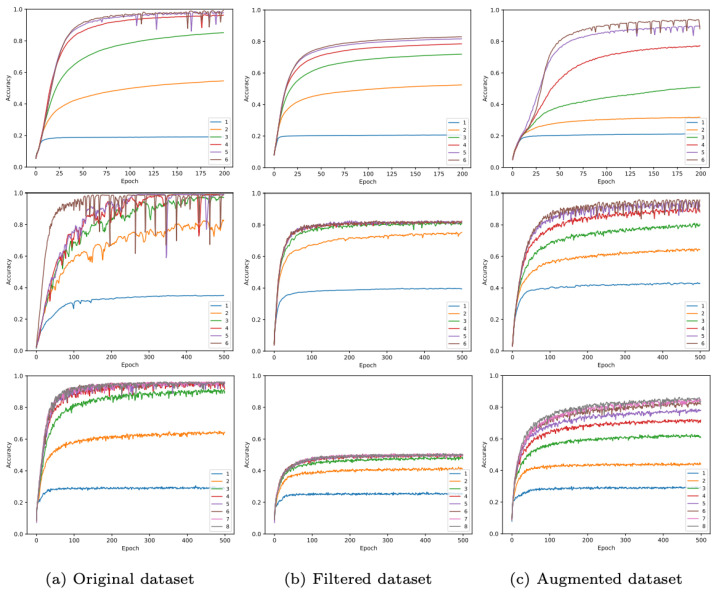
Training performance on the original, filtered, and augmented dataset for different numbers of layers in GNN. The first row is for the small-sized map where the best accuracies are reported to be 99.1%, 83.0%, and 94.0%, respectively; the second row is for the medium-sized map where the best accuracies are 98.9%, 82.7%, and 96.1%, respectively; and the bottom row is for the large-sized map, where best accuracies are 96.1%, 51.0%, and 87.5%, respectively.

The training process is completed on a desktop computer with GeForce GTX 1080. The Adam optimizer [58] is used to estimate the network weights. The hyperparameter settings are set to the following values: the application of 1 to 6 different GNN layers, the learning rate is set to 0.001, and the batch size is set to 512. The number of training epochs is set to 200 for the small-sized map and 500 for the medium- and large-sized maps. The training results are shown in Figure 8. It is observed that deeper graph layers tend to obtain higher accuracy and that augmented data apparently attain higher accuracy than filtered data. To be clear about the role of the training process, training accuracy can represent testing results in the sense that the only difference between training and testing is the map-generated trajectory and the real trajectory.

## 5. Results and Analyses

We have shown the training performance in Figure 8 and observe that the graph neural network is able to learn the representation of path subgraph for each class and perform node classification with satisfying accuracy. To evaluate the performance, we choose to use the accuracy metric, which is also consistent with the other latest works where the classification accuracy is a major evaluation metric [40,61,62]. The accuracy here is computed as:

(8) $$Accuracy\left(i\right)=\frac{1}{N}\sum_{i}^{N}S_{i}$$

where $S_{i}$ is the correctness of prediction {0,1} of path subgraph *i* and *N* is the total number of subgraphs. The reason why we only choose accuracy as an evaluation metric is twofold. First, our training dataset does not have an imbalance issue, which is the major cause of using another evaluation metric, such as recall and precision. Second, due to the special aspect of our task, which is geolocalization, the difference between testing and training datasets is only simulated map-generated and real data. Therefore, we only focus on what percentage of real data can be correctly classified. The training performance in the original dataset is only used as a reference. However, the best accuracy can reach up to around 99% in this case, which demonstrates the effectiveness of the proposed method. For the other two cases, it can be observed that the performance of the augmented dataset is better than the filtered dataset because the filtered dataset only contains turning information while the augmented dataset encodes both turning and distance information. For maps with different sizes, the less complex environment obtains the best accuracy among the three maps, showing that the performance of the model is relevant to the map size. The large map area carries the difficulty in generating all possible trajectories on that map and ambiguity between different trajectories. In the ablation study discussed later, we also show the accuracy as a function of the route length.

**Figure 9:**
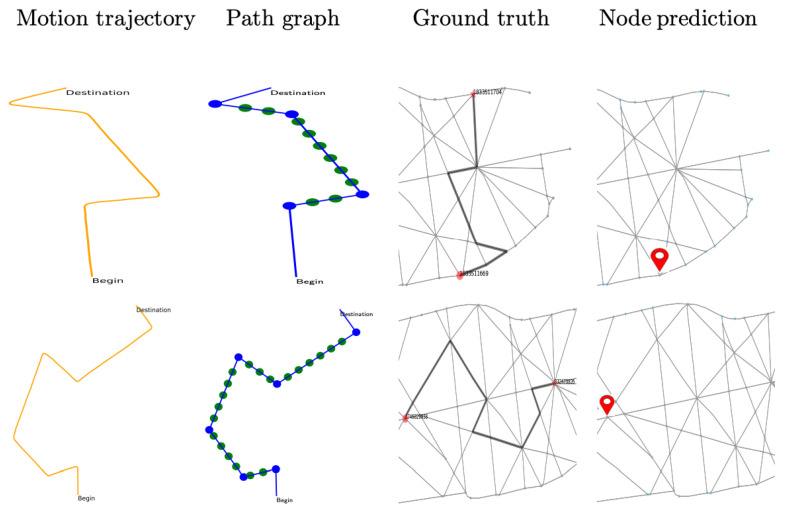
Testing results on real trajectories generated using visual-inertial odometry.

**Figure 10:**
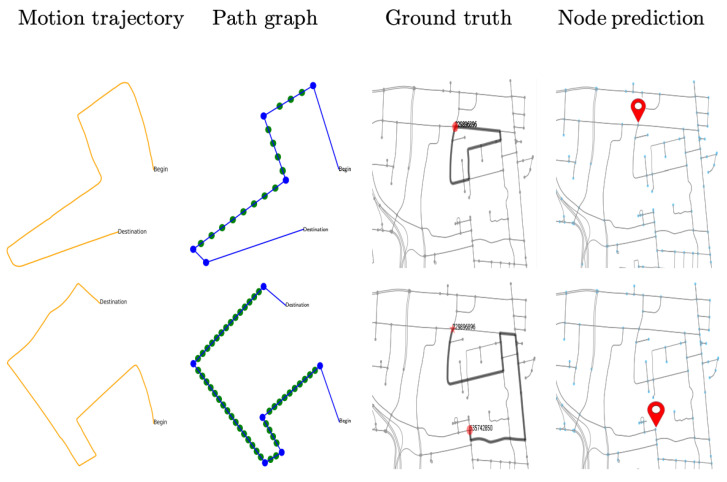
Testing results on real trajectories generated using visual-inertial odometry.

**Figure 11:**
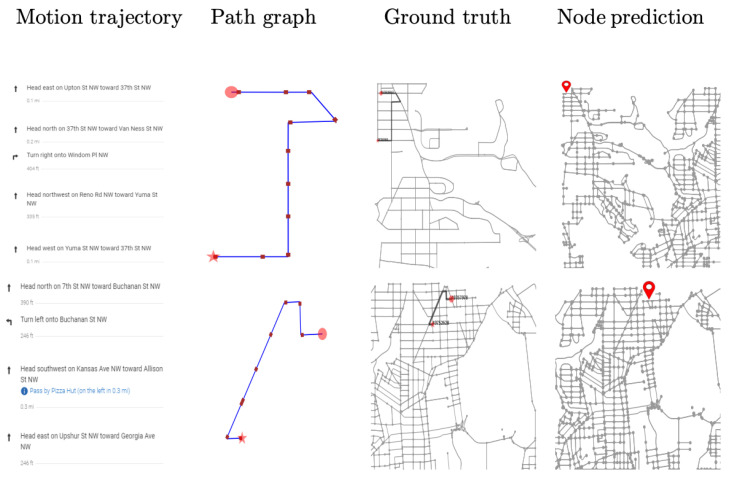
Testing results on real trajectories generated using visual-inertial odometry.

For generating testing trajectories, the raw data are first processed to construct the subgraph and tested using the trained network. The result is illustrated in Table 3, where the classification accuracy in the small-sized map is observed to be 85% for augmented representation, 90% in the medium-sizeddriving map, and 84% in a large-sized city map. The visual presentation is provided in Figure 9, Figure 10 and Figure 11 using six successful testing results, from which we should note that the quality of real trajectory affects the testing performances, while our approach is robust to noise due to discretization and sampling of virtual nodes. The failure cases during the testing are caused by two main factors. One is related to the training performance of the network, and the other one is related to the identification of the nodes where a turning occurs from the noisy trajectory generated using visual-inertial odometry.

### 5.1. Comparisons with Existing Methods

The proposed method is different from localization approaches in the published literature, and a direct comparison of the performance, especially in the context of platform navigation, is not possible; nonetheless, we include thematic and accuracy comparisons to several state-of-the-art localization approaches in Table 4 in a number of descriptive and quantitative aspects for the case when the accuracy measure is defined similarly to ours. The methods that use OSM [34,35,36] all adopt traditional probabilistic frameworks, which is complicated for modeling and inference. The work [7,63] solves localization in the navigation task using image-based deep learning, whereas ours is focused on localization alone based on the path subgraph. The two main studies [41,43] achieve close accuracy in a very small testing area, but our method is tested on a city-size map and can easily be extended to longer trajectories due to the message-passing mechanism of GNN.

The last approach [61] achieves around 90% geolocalization accuracy using map tile embedding and street view image, which takes advantage of a contrastive learning technique and is still subject to the constraints of image appearance, while our motion-based localization shows better accuracy. Overall, the proposed method implements novel motion-based geolocalization on the graph representation of a map without an initial position.

### 5.2. Ablation Study

We evaluated the model performance on a different number of nodes in the path subgraph in Table 5 and different graph convolution types in Table 6. It can be observed that the more nodes in the path subgraph, the higher the accuracy becomes. We see that the augmented path representation performs significantly better than the filtered path representation, which verifies the hypothesis that the last node location is unique when more path information is incorporated. The accuracy in the large-sized city map is observed to be lower than the other two maps due to the Manhattan-like map structure, leading to more ambiguous repeating patterns. Overall, the medium-sized map performs better than the other two. We analyzed two major factors that could lead to this phenomenon. Within the same network architecture, the complexity of map structure is higher for the small-sized map, as we showed in Table 1. Furthermore, the large-sized map has far more output labels than the other two, which can cause a decrease in performance. The experiments also show that in Table 6, the GraphSAGE model obtains better results than other architectures.

### 5.3. Discussions

Our work differs from existing image-based geolocalization methods and is the first study to achieve geolocalization using a GNN to the best of our knowledge. Although we have evaluated our approach in three different sizes of map datasets using different route lengths, it is still necessary to clearly elaborate on several concerns and limitations of the proposed method.

#### 5.3.1. Manhattan-World Ambiguity

The three maps are used in this article are all not equipped with repeated patterns. However, a few special road network structures exist, such as Manhattan-world or the lattice-structured environment, which pose a challenge to our motion-based method since a sequence of motions will correspond to multiple locations such as in the large-sized Washington D.C. map due to repeating trajectory patterns. However, there are a large number of one-way streets in a lattice-like road network, and a directed map graph can significantly reduce such ambiguity. The experiment on the Washington D.C. map shows the accuracy is still acceptable when the route length is increased, as shown in Table 5.

#### 5.3.2. Scalability

An important limitation of our method is the handling of significantly large map areas (>>100 km${}^{2}$). This is due to the increased label space causing problems at the softmax classifier layer. Nonetheless, to verify if the proposed method can extend to the city-scale area and to understand how the map size impacts the performance, we have studied three different sizes: small-sized (0.1 km${}^{2}$), medium-sized (6 km${}^{2}$), and large-sized (100 km${}^{2}$). The results show that the training accuracy in the large map is lower than the other two maps for the same trajectory window. However, the results still demonstrate an acceptable accuracy of around 85% for a 100 km${}^{2}$ region. We should also note that an increase in the path length as shown in the ablation study would reduce the ambiguity while increasing accuracy.

#### 5.3.3. Image as Complementary Data

Although the visual data are considered to be a crucial distinguishable feature, much of the world is ever-changing, and maintaining updated images will likely remain a challenge. Our experiments on small- and medium-sized maps show the accuracy is still promising even without visual data as shown in Figure 8. In lattice-like or Manhattan-world maps, the visual data would be helpful to some degree. Nonetheless, our work focuses on a pure motion-based approach where the motion data source is easy to fulfill in practice and is robust against changes in lighting and weather conditions across day times and seasons.

## 6. Conclusions

In this paper, we propose a subgraph learning and classification approach for topological geolocalization based on the platform’s motion, using a graph neural network. Instead of estimating the exact pose, our method provides a new perspective to address the problem of geolocalization by classifying the motion trajectory in the graph representation of a map node space. The training on map-generated data with two different subgraph representations on three different map sizes has performed positively, which suggests that the proposed neural network is able to achieve the geolocalization task. We also validate the effectiveness of our method on three real small-, medium-, and large-sized maps using the real-world trajectory.

## Figures and Tables

**Table 1:** The graph statistics for three different sizes of the map. The average degree centrality here is used to indicate the structure complexity of areas when generating possible paths.

	Location	Node	Edge	Map Size	Avg. Centrality
Small-sized map (S)	OSU Oval	91	155	0.16 km ∗ 0.5 km	3.5
Medium-sized map (M)	OSU Campus	115	147	2.5 km ∗ 2.5 km	2.54
large-sized map (L)	Washington DC	3038	8211	10 km ∗ 10 km	2.66

**Table 2:** Trajectory dataset statistics. Three different training datasets correspond to the three subgraph representations in Section 3.2. Here, Num. represents the total number of path subgraphs and Cls. represents the node classes.

	Original		Filtered		Augmented	
	Num.	Cls.	Num.	Cls.	Num.	Cls.
S	235,132	29	231,967	29	231,967	29
M	10,574	72	8551	72	8551	72
L	644,088	1000	644,088	1000	644,088	1000

**Table 3:** Real visual-inertial odometry trajectory testing result, including 20 trajectories for walking map and 10 trajectories for driving map.

	Filtered Case	Augmented Case
S: 20	14 (70%)	17 (85%)
M: 10	7 (70%)	9 (90%)
L: 50	25 (50%)	42 (84%)

**Table 4:** Descriptive and limited quantitative comparison with state-of-the-art methods for localization on driving map. Our method achieves better results with a topological representation that exploits graph neural networks. Note that “Metric” and “Non-Metric” indicate that the location is given by a numerical representation in a Cartesian coordinate system, and a non-numerical representation, such as a node or edge in a graph-structured map.

Method	Model	Map	Localization	Initial Position	NN	Input	Accuracy
2013 OpenStreetSLAM [35]	MCL	Graph	Metric	✓	✗	Image	∼5 m
2015 Brubaker et al. [34]	State-Space	Graph	Metric	✓	✗	Image	∼4 m
2017 Gupta et al. [36]	Graph Search	Graph	Metric	✗	✗	Image/IMU	∼5 m
2019 Amini et al. [63]	Variational NN	Tile	Metric	✓	✓	Image	−
2019 Chen et al. [7]	CNN+GNN	Graph	Non-metric	✓	✓	RGBD	−
2020 Wei et al. [41]	Seq2Seq	Graph	Non-metric	✗	✓	Motion	95%
2020 Zha et al. [43,64]	RNN	Graph	Non-metric	✗	✓	Motion	93%
2020 Samano et al. [61]	CNN	Tile	Non-metric	✗	✓	Image	90%
		Graph (S)					93.61%
**Ours**	GNN	Graph (M)	Non-metric	✗	✓	Motion	95.53%
		Graph (L)					87.56%

**Table 5:** The ablation study on training performance on different nodes of path subgraph in six layers using the GNN-SAGE model. It can be seen that the augmented dataset outperforms the filtered dataset and that the medium-sized map achieves the best accuracy.

Nodes	S		M		L	
	Filtered	Augmented	Filtered	Augmented	Filtered	Augmented
4	47.18%	66.71%	55.56%	81.48%	-	-
5	46.56%	67.72%	69.71%	88.24%	2.40%	10.70%
6	53.15%	72.82%	78.95%	89.65%	6.40%	24.70%
7	58.05%	77.05%	85.28%	91.48%	13.01%	40.12%
8	68.52%	89.47%	86.57%	93.20%	21.90%	58.42%
10	83.54%	93.61%	88.61%	95.33%	51.00%	87.50%

**Table 6:** The ablation study on training performance on different GNN models. As can be seen, the GNN-SAGE model outperforms the other models tested.

Model	S		M		L	
	Filtered	Augmented	Filtered	Augmented	Filtered	Augmented
**GNN-GCN**	75.31%	86.56%	82.04%	85.42%	49.91%	78.72%
**GNN-GAT**	71.39%	86.81%	82.63%	87.44%	49.85%	79.22%
**GNN-SAGE**	83.54%	**93.61%**	88.61%	**95.33%**	51.20%	**87.55%**
